# Eindrucksvolle Manifestation und unerwarteter Therapieverlauf eines Diabetes mellitus bei einem 22-jährigen Patienten

**DOI:** 10.1007/s00108-024-01797-x

**Published:** 2024-09-28

**Authors:** Christoph Werner, Sebastian Schmidt, Christiane Kellner, Katharina Burghardt, Philipp A. Reuken, Christof Kloos, Gunter Wolf

**Affiliations:** 1 Klinik für Innere Medizin III, FB Endokrinologie und Stoffwechselerkrankungen, Universitätsklinikum Jena, Am Klinikum 1, 07747 Jena, Deutschland; 2 Praxis für Humangenetik, Zentrum für ambulante Medizin, Universitätsklinikum Jena, Jena, Deutschland; 3 Klinik für Innere Medizin IV, Universitätsklinikum Jena, Jena, Deutschland

**Keywords:** „Maturity onset diabetes of the young“ (MODY), Diabetes mellitus, Sulfonylharnstoffe, Hyperglykämie, Glimepirid, Maturity onset diabetes of the young (MODY), Diabetes mellitus, Sulfonylurea receptors, Hyperglycemia, Glimepiride

## Abstract

Wir berichten über einen 22-jährigen Patienten, der nach einer längeren Remission ohne antidiabetische Therapie bei MODY („maturity onset diabetes of the young“) 12 (Gen *ABCC8*, nach ACMG-Kriterien Klasse 3) nach akuter Entgleisung und chronischer Hyperglykämie ein gutes Ansprechen auf Glimepirid zeigt. Wir geben einen kurzen Überblick über die pathogenetischen Grundlagen dieser genetischen Erkrankung und wollen das Bewusstsein für diese therapeutisch wichtige Entität schärfen.

## Abstract (en)

The case of a 22-year-old male patient who presented with acute on chronic hyperglycemia in known MODY („maturity onset diabetes of the young“) 12 (*ABCC8 *gene) after 11 months of treatment cessation is reported. To emphasize the importance of the awareness of this therapeutically important entity of diabetes, the essential facts of this inherited disease are summarized.

## Anamnese

Ein 22-jähriger Patient wird auf unsere endokrinologisch/diabetologische Station aufgenommen, nachdem er sich in unserer Hochschulambulanz vorgestellt hat. Aktuell berichtet er über Wohlbefinden, jedoch bemerkte er eine Polyurie und Polydipsie und einen Gewichtsverlust von 4 kg in 3 Monaten.

Seit ca. 2 Jahren wird er bei bekanntem Diabetes mellitus nach akuter ödematöser Pankreatitis ambulant von uns betreut. Die initiale Klassifikation bei Manifestation war Diabetes mellitus Typ 2 bei fehlendem Autoantikörpernachweis (GAD [Glutamatdecarboxylase], IAA [Insulinautoantikörper], ICA [Inselzellantikörper], IA2 [Tyrosinphosphatase 2], ZnT8 [Zinktransporter 8]), normwertigem C‑Peptid (3,0 ng/ml [NB: 1,1–4,4 ng/ml]) und adipösem Ernährungszustand (BMI 36,5 kg/m^2^). Das Alter des Patienten bei Manifestation war 19 Jahre.

Die letzte Vorstellung in unserer Ambulanz erfolgt 11 Monate vor der aktuellen Aufnahme. Im Anschluss hatte der Patient von sich aus seine bis dahin bestehende Therapie mit Metformin beendet, nachdem zuvor bereits die initial eingeleitete intensivierte konventionelle Insulintherapie bei guter glykämischer Kontrolle beendet werden konnte.

## Diagnostik

Aufgrund einer auffälligen Familienanamnese hatte sich der Patient auf unsere Empfehlung hin in der Humangenetik vorgestellt. In der Stammbaumanalyse ergaben sich folgende relevanten Befunde: Die Mutter des Patienten hatte einen Gestationsdiabetes, jetzt besteht ein Diabetes mellitus Typ 2. Die Großmutter (mütterlicherseits) hatte ebenfalls wahrscheinlich einen Diabetes mellitus Typ 2. Es erfolgte eine gezielte genetische Diagnostik bei der Mutter des Patienten auf die bei ihm festgestellte unklare Veränderung im Gen *ABCC8*, welche anamnestisch nicht nachgewiesen wurde. Weiterhin gibt es 3 Geschwister, bisher ohne bekannten Diabetes mellitus.

Es wurde dem Patienten zur Konkretisierung seiner Situation die Möglichkeit der molekularen Diagnostik hinsichtlich eines MODY („maturity onset diabetes of the young“)-Diabetes (Panel-Diagnostik mit 14 Genen) angeboten. Diese erfolgte über EDTA-Blut. Hier wurde die Diagnose MODY Typ 12 gesichert mit Nachweis einer heterozygoten Sequenzvariante im Gen *ABCC8* (c.1252T > C p. [Cys418Arg]). Die Panel-Diagnostik (14 Gene) erfolgte über Library Preparation mittels Target Enrichment (custom, TWIST Bioscience, South San Francisco, CA, USA) und Sequenzierung (NGS) auf der Illumina Plattform (NextSeq1000, San Diego, CA, USA). Die Analyse der NGS-Daten wurde mit der bioinformatischen Software varvis®, Version 1.21 (Limbus Medical Technologies GmbH, Rostock, Deutschland), bewertet. Für 100 % der untersuchten Genabschnitte (proteincodierende Exons inklusive der Exon-Intron-Grenzen ±30 bp) wurde eine mindestens 20fache Sequenziertiefe („coverage“) erreicht. Die molekulargenetische Diagnostik erbrachte im Exon 8 des Gens *ABCC8* den Nachweis einer heterozygoten Sequenzvariante des Codons 418 (CTC>CCC). Es handelt sich nach den American-College-of-Medical-Genetics-and-Genomics(ACMG)-Kriterien um eine Variante unklarer Signifikanz (Befund vom 06.04.2023, MVZ Labor Dr. Limbach Heidelberg). Diese Variante ist in der uns zugänglichen Literatur bisher noch nicht beschrieben. Eine Analyse mit dem Clinical Insight-System der Fa. Qiagen (Hilden, Deutschland) kommt zu dem Ergebnis, dass es sich nach ACMG-Kriterien um eine Variante unklarer Signifikanz handelt. Die Klassifizierung dieser Varianten basiert auf den ACMG-(ACGS-2020v4.01‑)Richtlinien [9]. Für die Beurteilung der Varianten wurden die klinischen Informationen herangezogen, die zum Zeitpunkt der Auswertung vorlagen. Befundet werden nur Varianten, die entsprechend der aktuellen Datenlage nicht als benigne oder wahrscheinlich benigne eingestuft wurden.

Die weiteren untersuchten Gene (*HNF-4A, GCK, HNF-1A, PDX1, HNF-1B, NEURO D1, KLF11, CEL, PAX4, INS, BLK, KCNJ11, APPL1*) waren unauffällig.

**Abb. 1:**
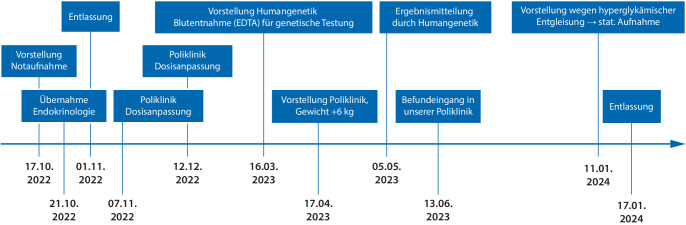
Zeitstrahl mit Darstellung des Verlaufs von Diagnostik und Therapie

Dieser Befund erging jedoch an uns leider erst nach der letzten Vorstellung in unserer diabetologischen Poliklinik (siehe Abb. 1).

## Befund

Blutdruck 162/104 mm Hg (Spontanmessung im Sitzen mit elektronischem Messgerät mit passender Oberarmmanschette), HF 80/min, Körpergröße 179 cm, Gewicht 117 kg, BMI 34,9 kg/m^2^. In der klinischen Untersuchung zeigten sich keine Auffälligkeiten, insbesondere keine Xanthome oder Xanthelasmen. Klinischer Anhalt für eine erneute Pankreatitis bestand nicht, ebenso keine klinischen Hinweise auf Hormonexzess oder -mangel.

Im EKG fand sich ein altersentsprechender Normalbefund.

Das HbA1c lag bei 13,4 % (119 mmol/mol; NB: < 5,7 %, 38,8 mmol/mol, Messung mittels HPLC), die Nüchternplasmaglukose bei 10,6 mmol/l, postprandial bei 18,8 mmol/l (jeweils kapillär gemessen mit POCT-Gerät [elektrochemisch, Glukoseoxidase-Methode] der Klinik). Im Urin zeigten sich die Ketonkörper 3fach positiv (Urine Chemistry Strips mit iRIS iChem Velocity von Iris Diagnostic Division, Chatsworth, CA, USA). Die venöse BGA zeigte einen ausgeglichenen pH (7,35 [7,35–7,45]) mit normwertigem Bikarbonat (24,6 mmol/l [21–26 mmol/l]) und normwertigem Base-Excess (1,6 mmol/l [NB: –2,0–3,0 mmol/l]). Die Elektrolyte befanden sich im Normalbereich (Natrium 142 mmol/l [135–145 mmol/l], Kalium 4,2 mmol/l [3,3–4,5 mmol/l], Chlorid 98 mmol/l [98–107 mmol/l], ion. Kalzium 1,22 mmol/l [1,15–1,29 mmol/l]), das Laktat war nicht über den Normalbereich erhöht (1,5 mmol/l [NB: 0,5–2,2 mmol/l]). Kreatinin (96 µmol/l [62–106 µmol/l]), eGFR nach CKD-EPI-Formel (96,3 ml/min/1,73 m^2^ [NB: >90]) und Blutbild waren ebenfalls im Normalbereich, es bestand eine Hypertriglyzeridämie von 24,8 mmol/l (NB: <1,7 mmol/l). Die Serumlipase lag im methodenspezifischen Normalbereich.

## Therapie und Verlauf

Nach parenteraler Volumensubstitution und Beginn einer subkutanen Insulintherapie mit Insulin aspart (13 IE an Tag 1) sowie der Wiederaufnahme der Therapie mit Metformin zeigte sich ein ebenfalls bestehender Bedarf an Basalinsulin aufgrund von Blutglukoseanstiegen über Nacht, welcher mit Insulin glargin abgedeckt wurde (37 IE an Tag 2 für insg. 8 KE). Nach rascher Normalisierung der Blutglukose unter 10 mmol/l beendeten wir das Mahlzeiteninsulin und ergänzten den Sulfonylharnstoff Glimepirid (1 mg 3‑mal/d zur Mahlzeit). Diesen wählten wir aufgrund der kurzen Halbwertszeit bei wechselnden Tagesabläufen und Mahlzeiteneinnahmen im Rahmen eines Schichtdienstes des Patienten. Trotz einer Dosissteigerung des Metformins auf 2‑mal 1000 mg/d blieb bis zur Entlassung ein Bedarf an Basalinsulin von 10 IE zur Nacht bestehen.

Während des stationären Aufenthalts nahm der Patient an einem curricularen, strukturierten Schulungs- und Behandlungsprogramm durch unsere Diabetesberaterinnen teil, mit Inhalten zur Blutglukoseselbstkontrolle, Ernährung, Therapieanpassung und Behandlung von Komplikationen.

Unter der oben beschriebenen Therapie und der Normalisierung der Hyperglykämie sanken die Triglyzeride sukzessive bis auf 7,07 mmol/l ab. Als Auffälligkeit im Labor nahm das Bilirubin bis 35 µmol/l (NB: < 21 µmol/l) zu. Wir veranlassten eine Abdomensonographie und konnten eine Cholestase sicher ausschließen. Es fanden sich jedoch eine Hepatomegalie bei hypertropher Fettleber sowie eine Pankreaslipomatose.

Wir entließen den Patienten mit Blutglukosewerten im Zielbereich der nahen Normoglykämie (Nüchternglukose 6,4 mmol/l). Ein Folgetermin in unserer Poliklinik zur Therapiekontrolle ist bei Manuskripterstellung ausstehend.

## Diskussion

Der „maturity onset diabetes of the young“ (MODY) ist eine Unterform des Diabetes mellitus und wird auf eine Prävalenz von ca. 1–2 % der Diabeteserkrankungen in Europa geschätzt und wird meist im Kindes- oder frühen Erwachsenenalter manifest. Ein MODY wird autosomal-dominant vererbt (Vererbungswahrscheinlichkeit an Kinder eines Betroffenen 50 %), die Störung erfolgt durch eine Veränderung in Genregionen, die für den Glukosestoffwechsel bedeutsam sind. Seit der klinischen Erstbeschreibung 1974 konnten durch verbesserte Verfahren und leichter verfügbare genetische Diagnostik 14 (Stand 2022; [10]) Genmutationen identifiziert werden. Die Mutationen verursachen meist Störungen in der Promoter-Region des Insulingens oder des ATP-abhängigen Kaliumkanals. Am häufigsten treten MODY 3 (Gen *HNF1A*, 30–50 %), MODY 2 (Gen *GCK*, 15–20 %), MODY 1 (Gen *HNF4a*, 5 %) und MODY 5 (Gen *HNF1B*, 5 %) auf. Die weiteren Formen werden mit <1 % der MODY-Fälle angegeben (siehe Tab. 1; [1]).

**Tab. 1:** Übersetzt aus Referenz [4], adaptiert nach [8]

MODY	Gen	Chromosomenabschnitt	Häufigkeit (Anteil an MODY, %)	Pathophysiologie	Weitere Merkmale	Therapie
1	*HNF4A*	20q13	5	Β‑Zell-Dysfunktion	Neugeborenenhypoglykämie, niedrige Triglyzeride	Sulfonylharnstoffe
2	*GCK*	7p13	15–20	Β‑Zell-Dysfunktion (Glukosesensordefekt)	Nüchternhyperglykämie des Neugeborenen	Adaptierte Kost
3	*HNF1A*	12q24	30–50	Β‑Zell-Dysfunktion	Glukosurie	Sulfonylharnstoffe
4	*PDX1/IPF1*	13q12	<1	Β‑Zell-Dysfunktion	Pankreasagenesie bei Homozygotie	Adaptierte Kost, OAD oder Insulin
5	*HNF1B*	17q12	5	Β‑Zell-Dysfunktion	Renale und genitale Fehlbildungen, hypoplastisches Pankreas	Insulintherapie
6	*NEUROD1*	2q31	<1	Β‑Zell-Dysfunktion	Diabetes im Erwachsenenalter	OAD oder Insulin
7	*KLF11*	2p25	<1	Β‑Zell-Dysfunktion	Klinisch wie Diabetes mellitus Typ 2	OAD oder Insulin
8	*CEL*	9q34	<1	Endo- und exokrine Pankreasdysfunktion	Exokrine Pankreasinsuffizienz, Lipomatose	OAD oder Insulin
9	*PAX4*	7q32	<1	Β‑Zell-Dysfunktion	Ketoazidose möglich	Adaptierte Kost, OAD oder Insulin
10	*INS*	11p15	<1	Mutation Insulingen	Möglicher angeborener Diabetes	OAD oder Insulin
11	*BLK*	8p23	<1	Defekt Insulinsekretion	Übergewicht, rel. Insulinsekretionsdefekt	Adaptierte Kost, OAD oder Insulin
12	*ABCC8*	11p15	<1	Defekt des ATP-abhängigen Kaliumkanals	Angeborener Diabetes, persistierend bei Homozygotie	OAD, (Sulfonylharnstoffe)
13	*KCNJ11*	11p15	<1	Defekt des ATP-abhängigen Kaliumkanals	Angeborener Diabetes bei Homozygotie	Adaptierte Kost, OAD oder Insulin
14	*APPL1*	3p14.3	<1	Adapterprotein im Insulinsignalweg	Relativ späte Manifestation, variable Penetranz	Adaptierte Kost, OAD oder Insulin

*MODY* „maturity-onset diabetes of the young“, *NF4A* „hepatocyte nuclear factor 4α“, *GCK* „glucokinase“, *PDX1* „pancreatic and duodenal homeobox 1“, *IPF1* „insulin promoter factor 1“, *OAD* „oral antidiabetic agents“, *NEUROD1* „neurogenic differentiation 1“, *KLF11* „Kruppel-like factor 11“, *CEL* „carboxyl ester lipase“, *PAX4* „paired-box-containing gene 4“, *INS* „insulin“, *BLK* „B-lymphocyte kinase“; *ABCC8* „ATP-binding cassette, subfamily C (CFTR/MRP), member 8“; *ATP* „adenosine triphosphate“; *KCNJ11* „potassium channel, inwardly rectifying subfamily J, member 11“, *APPL1* „adaptor protein, phosphotyrosine interaction, PH domain, and leucine zipper containing 1“

Der MODY Typ 12 ist durch eine Mutation im Gen *ABCC8* („ATP-binding cassette, subfamily C [CFTR/MRP], member 8“) charakterisiert, welches für die Untereinheit (SUR 1) codiert, die den Angriffspunkt für Sulfonylharnstoffe im ATP-abhängigen Kaliumkanal der pankreatischen Betazelle darstellt. Der MODY 12 stellt mit einem Anteil <1 % eine der seltenen Formen dar.

Pathogene Sequenzvarianten im Gen *ABCC8* gehen mit MODY Typ 12 einher. Der sich oft innerhalb der ersten sechs Lebensmonate manifestierende permanente neonatale Diabetes mellitus (PNDM) wird auch durch Mutationen im Gen *ABCC8* verursacht. PNDM folgt bei Veränderung im Gen *ABCC8* sowohl einem autosomal-dominanten als auch einem autosomal-rezessiven Vererbungsmodus [2, 3, 5–7].

Bei homozygoten Trägern des MODY 12 tritt bereits im Neugeborenenalter ein Diabetes mellitus auf, während sich die heterozygote Mutation später manifestiert.

Andere Formen des MODY gehen ebenfalls mit neonatalem Diabetes einher (MODY 10, MODY 13), wieder andere mit kongenitalen Anomalien oder gehäuften Komplikationen (MODY 4 – Pankreasagenesie; MODY 5 – Nieren- und Genitalfehlbildungen, Pankreashypoplasie; MODY3 – Neigung zu mikrovaskulären Folgeerkrankungen ähnlich wie bei Diabetes mellitus Typ 1 [4]).

Pathophysiologisch kann durch die Therapie mit Sulfonylharnstoffen eine verstärkte Aktivierung des entsprechend funktionsgeminderten Kanals erreicht werden, wodurch die mutationsbedingt verminderte Insulinsekretion ausgeglichen werden kann. In der Literatur wird beschrieben, dass durch diese Wirkstoffgruppe oft eine langjährige Therapie möglich erscheint und diese somit die Therapie der ersten Wahl darstellt (MODY 1, 3, 12; [4]).

Unter Metformin gelingt, wie im konkreten Fallbeispiel zu sehen, häufig eine gute Therapiekontrolle. Hier ist, wie auch in dem von uns beschriebenen Fall, unter anderem von einer verbesserten Insulinwirkung bzw. Reduktion der Resistenz auszugehen.

Der MODY 2 kann häufig mit einer kohlenhydratarmen Ernährung behandelt werden, eine Insulintherapie wird hier nur bei Schwangerschaft oder homozygoten Trägern notwendig [4].

Liegt eine familiäre Häufung von Diabetes mellitus vor und erfolgt die Manifestation im jungen Erwachsenenalter, ist der Patient schlank oder hat trotz normaler Blutglukose eine Glukosurie, sollte ein MODY in die Differenzialdiagnose einbezogen werden.

## Fazit für die Praxis

Der MODY, welcher heute treffender als monogenetischer Diabetes zu bezeichnen wäre, stellt eine unterdiagnostizierte Krankheitsentität dar. Die Diagnose ist essenziell, da sich aus der Pathophysiologie der Erkrankung wichtige, für den Patienten vorteilhafte, Behandlungskonzepte ergeben. Zudem ist eine humangenetische Beratung für die Familienplanung und ggf. frühzeitige Therapie zur Vermeidung von Folgeerkrankungen unerlässlich.

## References

[1] Aarthy R, Aston-Mourney K, Mikocka-Walus A et al (2021) Clinical features, complications and treatment of rarer forms of maturity-onset diabetes of the young (MODY) – A review. J Diabetes Complications 35:107640 32763092 10.1016/j.jdiacomp.2020.107640

[2] Broome DT, Pantalone KM, Kashyap SR et al (2021) Approach to the Patient with MODY-Monogenic Diabetes. J Clin Endocrinol Metab 106:237–250 33034350 10.1210/clinem/dgaa710 PMC7765647

[3] Delvecchio M, Pastore C, Giordano P (2020) Treatment Options for MODY Patients: A Systematic Review of Literature. Diabetes Ther 11:1667–1685 32583173 10.1007/s13300-020-00864-4 PMC7376807

[4] Kim SH (2015) Maturity-Onset Diabetes of the Young: What Do Clinicians Need to Know? Diabetes Metab J 39:468–477 26706916 10.4093/dmj.2015.39.6.468 PMC4696982

[5] Ludvigsson J, Carlsson A, Forsander G et al (2012) C‑peptide in the classification of diabetes in children and adolescents. Pediatr Diabetes 13:45–50 21910810 10.1111/j.1399-5448.2011.00807.x

[6] Naylor R, Knight JA, Del Gaudio D (1993) Maturity-Onset Diabetes of the Young Overview. In: Adam MP, Feldman J, Mirzaa GM, Pagon RA, Wallace SE, Bean LJH, Gripp KW, Amemiya A (Hrsg) GeneReviews, Seattle 29792621

[7] Nkonge KM, Nkonge DK, Nkonge TN (2020) The epidemiology, molecular pathogenesis, diagnosis, and treatment of maturity-onset diabetes of the young (MODY). Clin Diabetes Endocrinol 6:20 33292863 10.1186/s40842-020-00112-5 PMC7640483

[8] Prudente S, Jungtrakoon P, Marucci A et al (2015) Loss-of-Function Mutations in APPL1 in Familial Diabetes Mellitus. Am J Hum Genet 97:177–185 26073777 10.1016/j.ajhg.2015.05.011 PMC4571002

[9] Richards S, Aziz N, Bale S et al (2015) Standards and guidelines for the interpretation of sequence variants: a joint consensus recommendation of the American college of medical genetics and genomics and the association for molecular pathology. Genet Med 17:405–424 25741868 10.1038/gim.2015.30 PMC4544753

[10] Tosur M, Philipson LH (2022) Precision diabetes: Lessons learned from maturity-onset diabetes of the young (MODY). J Diabetes Investig 13:1465–1471 35638342 10.1111/jdi.13860 PMC9434589

